# Transcriptome Characterization of Developing Bean (*Phaseolus vulgaris* L.) Pods from Two Genotypes with Contrasting Seed Zinc Concentrations

**DOI:** 10.1371/journal.pone.0137157

**Published:** 2015-09-14

**Authors:** Carolina Astudillo-Reyes, Andrea C. Fernandez, Karen A. Cichy

**Affiliations:** 1 Department of Plant, Soil and Microbial Sciences, Michigan State University, 1066 Bogue St., East Lansing, MI, United States of America; 2 USDA-ARS, Sugarbeet and Bean Research Unit and Department of Plant, Soil and Microbial Sciences, Michigan State University, 1066 Bogue St., East Lansing, MI, United States of America; National Institute of Plant Genome Research, INDIA

## Abstract

Dry bean (*Phaseolus vulgaris* L.) seeds are a rich source of dietary zinc, especially for people consuming plant-based diets. Within *P*. *vulgaris* there is at least two-fold variation in seed Zn concentration. Genetic studies have revealed seed Zn differences to be controlled by a single gene in two closely related navy bean genotypes, Albion and Voyager. In this study, these two genotypes were grown under controlled fertilization conditions and the Zn concentration of various plant parts was determined. The two genotypes had similar levels of Zn in their leaves and pods but Voyager had 52% more Zn in its seeds than Albion. RNA was sequenced from developing pods of both genotypes. Transcriptome analysis of these genotypes identified 27,198 genes in the developing bean pods, representing 86% of the genes in the *P*. *vulgaris* genome (v 1.0 DOE-JGI and USDA-NIFA). Expression was detected in 18,438 genes. A relatively small number of genes (381) were differentially expressed between Albion and Voyager. Differentially expressed genes included three genes potentially involved in Zn transport, including zinc-regulated transporter, iron regulated transporter like (ZIP), zinc-induced facilitator (ZIF) and heavy metal associated (HMA) family genes. In addition 12,118 SNPs were identified between the two genotypes. Of the gene families related to Zn and/or Fe transport, eleven genes were found to contain SNPs between Albion and Voyager.

## Background

Zinc is essential to human health and nutrition. Zinc is an important enzyme cofactor and component of proteins, and is needed for DNA synthesis, RNA transcription, and cell division [1]. Human Zn deficiency symptoms are quite varied, including reduced immune function, fetal brain cell development, and reproductive and cognitive development [2]. Mild to moderate Zn deficiency is common, especially in populations consuming vegetarian diets rich in unrefined cereals [3]. Biofortification of staple foods such as wheat and dry beans with Zn is one agricultural science based approach being developed and applied to combat micronutrient malnutrition [4].

Dry beans (*Phaseolus vulgaris* L.) are a nutrient dense food crop and a dietary staple in East Africa and Latin America. Genotypic variability for seed Zn levels is relatively high within the species and Zn seed levels from 20 to 59 μg g^-1^ have been observed [5,6]. Understanding the genetic control of seed Zn content has the potential to improve the breeding process for this important nutritional trait by identifying candidate genes for marker assisted selection and also increase the overall Zn content levels achievable through breeding.

Numerous genes involved in Zn transport have been characterized in model plant species including *Arabidopsis* and *Medicago* [7]. Major gene families shown to play a role in transport of Zn include, ZIP, YSL, NAS, ZIF, HMA and MTP. The ZIP family is made up of ZRT (zinc related transporter) and IRT (iron related transporter) like proteins. The common feature of members of this family is eight transmembrane domains and a metal binding domain [8]. In addition, transcription factors that regulate ZIP genes include members of the basic region leucine zipper (bZIP) gene family. bZIP19 and bZIP23 genes have been shown to interact with ZIP genes in *Arabidopsis* [9]. Yellow stripe like (YSL) is a gene family that transport metal-NA complexes long distance. In *Arabidopsis At*YLS*2* is responsible for mobilization of micronutrients such as Mn, Zn, Cu and Fe from leaves and for loading of Fe-NA complex into seed [10]. Nicotianamine (NA) a non proteinogenic amino acid chelates Fe and Zn phloem movement to sink tissue [11]. Four NA synthase genes have been characterized [12] and are related in reproduction and seed Fe loading [13]. Zinc sequestration and detoxification occurs in the vacuoles when zinc is abundant providing a site of zinc storage [14–16]. Several gene transporters have been reported to be involved in zinc translocation to the vacuoles. Zinc induced facilitator (ZIF1) protein contributes to Zn and NA sequestration into the vacuoles thus removing the opportunity for both to be transported symplastically [17]. Two members of the heavy metal ATPase gene family (HMA) HMA2 and HMA4, mediate the release of zinc in root-to-shoot transport of zinc into the xylem [8,18]. Two members of the cation diffusion facilitator (CDF) family, the metal tolerance protein (MTP) MTP1 and MTP3, have been found to be involved in vacuolar zinc sub-cellular sequestration and contribute to zinc tolerance in *A*. *thaliana* [19,20]. NRAMP (natural resistance associated macrophage protein) are involved in transport of metals out of vacuoles [21]. Six members have been identified in *Arabidopsis*. NRAMP3 and NRAMP4 are involved in iron remobilization from the vacuole [22]. In a yeast complementation analysis only NRAMP4 was able to complement *zrt1zrt2* growth under low zinc levels [23].

These gene families involved in mineral transport and sequestration represent some of the most obvious candidate genes for increased Zn seed levels in crops such as *P*. *vulgaris*. Very little is known about how Zn is transported from leaf xylem to phloem of developing seeds and ultimately unloaded into seeds [24].

Here we characterize the transcriptome of developing pod of two bean genotypes. These genotypes were shown to have similar Zn concentration in roots sampled during vegetative growth, leaves, and pods, but different levels of Zn in the seed. A total of 381 genes were differentially expressed including four genes that may play a role in Zn or other mineral transport to seeds including zinc-regulated transporter, iron regulated transporter like (ZIP), bZip transcription factor, zinc-induced facilitator (ZIF) and natural resistance associated macrophage protein (NRAMP) family genes. A total of eleven genes in the ZIF, NRAMP, YSL, and ferritin gene families contained SNPs between the two genotypes.

## Materials and Methods

### Plant Material

The two common bean genotypes used for this study both are navy beans from the Mesoamerican gene pool. Albion is a navy bean variety released by Asgrow in 1987, Voyager, is a navy bean released by Rogers Seed Company in 1995. These genotypes were selected based on their contrasting seed Zn concentration. Voyager has higher levels of seed Zn than Albion in diverse growing conditions [25]. In addition, in contrast to Voyager, Albion exhibits foliar Zn deficiency symptoms in low Zn and/or calcareous soils [26].

### Plant Zinc Uptake Experiment

Seeds of Albion and Voyager were individually planted in 500 ml pots with 3:1 Sunshine Brand premium grade vermiculate and horticultural perlite grade (P.V.P. Industries, Inc). Treatments consisted of 0.5X strength modified Hoagland solution [27] with zinc added and without zinc (3 mMKNO_3_, 2 mM Ca (NO_3_)_2_ x 4H_2_O, sequestrene DTPA 10% Fe, 1.0 mM MgSO_4_ x 7H_2_O, 23.1 mM H_3_BO_3_, 0.38 mM ZnSO_4_ x 7H_2_O, 0.16 mM CuSO_4_ x 5H_2_O, 4.6 mM MoO_4_ x 2H_2_O, 1M KH_2_PO_4_ (pH to 6.0) was used as a fertilization treatment at a rate of 400 ml three times per week. Plants were grown in a growth chamber with a photoperiod of 16 hours light and 8 hours dark. Two replicates per plant were harvested as follows: roots and leaf tissue samples of vegetative plants were collected when the third trifoliate leaf had unfolded. Tissue of roots and leaves during flowering was collected when 30% of flowers were opened. Flowering was monitored daily and pods were collected 20 d after flowering. Seed was collected at physiologic maturity. Tissue was collected in liquid nitrogen and stored at -80°C. All samples were lyophilized and ground to powder with a Geno/Grinder 2000 (SpexCertiPrep, Metuchen, NJ) and zircon grinding balls. Plant tissue samples sent to A & L Laboratories (Fort Wayne, IN) for mineral analysis using induced coupled plasma spectroscopy. Mineral concentration was measured on 48 beans samples as follows: six tissue types, two Zn fertilization treatments, two genotypes, and two replications of each. Statistical significance was determined using proc glm and Tukey tests for pairwise comparisons in SAS for Windows v.9.2 (SAS Institute Inc., Cary, NC, USA).

### Greenhouse RNA Seq Experiment

Seed of Voyager and Albion were planted in a greenhouse at Michigan State University. Two seeds were planted in 10 cm diameter clay pots filled with SureMix potting soil (Michigan Peat Company). Three pots were planted of each genotype and each pot was treated as a replication. Plants were watered as needed and fertilized with 0.5X Hoaglands solution (3 mM KNO_3_, 2 mM Ca (NO_3_)_2_ × 4H_2_O, sequestrene DTPA 10% Fe, 1.0 mM MgSO_4_ × 7H_2_O, 23.1 mM H_3_BO_3_, 0.38 mM ZnSO_4_ × 7H_2_O, 0.16 mM CuSO_4_ × 5H_2_O, 4.6 mM MoO_4_ × 2H_2_O, 1M KH_2_PO_4_ (pH to 6.0) biweekly starting at 20 d after germination. At anthesis, flowers were marked with a tag. At 12 days after anthesis individual pods were removed from plants and flash frozen in liquid nitrogen. Two pods per replication were ground to a fine powder with a mortar and pestle while completely frozen. Liquid nitrogen was continuously added to ensure tissue remained frozen throughout the grinding process. Total RNA was extracted from the samples using an RNA easy Plant kit (Qiagen, Cat. No. 74904). Following extraction, RNA samples were treated with RNase free DNase I (Qiagen Cat. No. 74254). RNA integrity and concentration was assessed for each of the samples using an Aligent 2100 Bioanalyzer (Agilent Technologies, Inc.). A subsample of the pod tissue was retained for mineral analysis. Following pod sampling, plants were grown to maturity and mature seeds were also analyzed for mineral concentration as described above and nitrogen concentration according to the Dumas method. Statistical significance was determined using proc glm and Tukey tests for pairwise comparisons (P<0.05) in SAS for Windows v.9.2 (SAS Institute Inc., Cary, NC, USA).

### RNA Sequencing and Pre-Processing

Six RNA samples in total (3 replicates each of Albion and Voyager) were sequenced at the Michigan State University Research Technology Support Facility (RTSF) using an Illumina Genome Analyzer II (GA II). The library and flow cell preparation using kits and protocols from Illumina was conducted by the MSU RTSF. The sequencing was conducted as 75-bp paired-end reads. The RNA sequence was received from RTSF in FASTQ formatted files containing 75-bp paired-end reads. The file contained sequences and quality information about each sequence. The data were filtered using scripts from FASTX-Toolkit (FASTQ Quality Trimmer and FASTQ Quality Filter http://hannonlab.cshl.edu/fastx_toolkit/). FASTQ Quality Trimmer clipped the low quality ends with a quality threshold of 20 and removed the reads shorter than 64 bp. Subsequently, FASTQ Quality Filter script was used to remove low quality sequences with quality scores of 20 or less.

The *P*. *vulgaris* reference genome sequence v. 1.0 (DOE-JGI and USDA-NIFA and corresponding gene model annotation files were obtained directly from http://www.phytozome.net) [28]. A *P*. *vulgaris* genome index was built using Bowtie v. 0.12.7 [29]. Paired-end clean reads for each individual were aligned to the reference genome by TopHat v 1.4.1 [30] with the provided guidance of annotated gene models (GTF file). Bam files for each alignment are deposited in Gene Expression Omnibus (GEO).

### Gene Ontology Enrichment Analysis

Mapped reads were evaluated for homology and annotated using Blast2GO software (http://www.blast2go.com). This process included three steps: 1) BLAST to find homologous sequences, with the following options, e-value threshold of E-^10^, non-redundant protein database (nr), high-scoring segment pairs (HSP) length cutoff 33. 2) MAPPING to retrieve (Gene ontology) GO terms and 3) ANNOTATION to select reliable functions, with e-value hit filter of 1E-6, cutoff 55, GO weight 5, Hsp-Hit coverage cutoff 0BLASTx sequence translation tool. We evaluated the functional enrichment level using the David Bioinformatics Database [31]. Significance of the enrichment analysis was declared when *p-value*<0.05.

### Differential Expression Analysis

Abundance estimation was carried out using Cufflinks v1.3.0 with default parameters [32]. Normalization, estimated abundance, and tests for differential expression between tissue samples were performed using the program Cuffdiff with default parameters. The value used to compute significance of the observed change of transcript abundance was measured in fragments per kilobase of transcript per million mapped reads (FPKM) [33]. The differentially expressed genes were reported as fold change (on a log_2_ scale), and significance in terms of *p*-value and *q*-values. FPKM greater than zero was used as the threshold to consider a gene as being expressed. The significance of the gene expression difference was determined when the *p*-value was lower than the false discovery rate (*q-value*) (FDR<0.05). To validate the accuracy of expression profiles obtained by RNAseq, RT qPCR was performed on 6 genes with high or low expression levels as described in the supporting information. The genes belong to the ZIP gene family and the bZIP transcription factors involved in zinc homeostasis [34].

### SNP Discovery and Validation

Single nucleotide polymorphisms between Voyager and Albion were discovered. Samtools mpileup [35] and bcftools were used for discovery and filtering using the following parameters: Alignments with a unique location, mapping quality >30, minimum depth = 8 and presence of SNP in >90% of reads on both forward and reverse strand. The options selected were–D (Output per-sample read depth),–u (Compute genotype likelihoods), and–f (The faidx-indexed reference file in the FASTA format). To validate SNPs called from the transcriptome sequence analysis, three genes YSL, HMA and ZIF were selected based on their role in Zn transport and presence of more than three SNPs. Primers were designed to amplify a template approximately 700 bp long. DNA was extracted from primary leaf tissue of Voyager and Albion using centrifugal filter “DNeasy Plant Kit” (QIAGEN) and quantified with Quant-iTPico Green dsDNA Assay kit (Invitrogen) following the manufacturer’s instructions. The mixture for each gene was optimized to contain 30 ng of DNA extract, 30 pmol of the primers and 0.5 U of Taq polymerase (AccuPrimePfxSuperMix, Invitrogen). After initial denaturation (95°C 5 min) 35 cycles (95°C 30 sec, 63°C 30 sec, 72°C 30 sec) of amplification were performed, followed by a final extension of 72°C for 5 min. Amplification products of both parents Albion and Voyager were visualized by electrophoresis on 2% agarose gel, stained with ethidium bromide and detected by ultraviolet trans illumination. PCR products with a single band were purified by ethanol precipitation, and directly sequenced via Sanger sequencing with the same primers used for PCR. Sequencing was conducted at the MSU RTSF. Sequenced products were compared and aligned with the reference common bean genome using Geneious (v. 5.6.2) (Biomatters).

## Results and Discussion

### Growth Chamber Experiment

**Fig 1:**
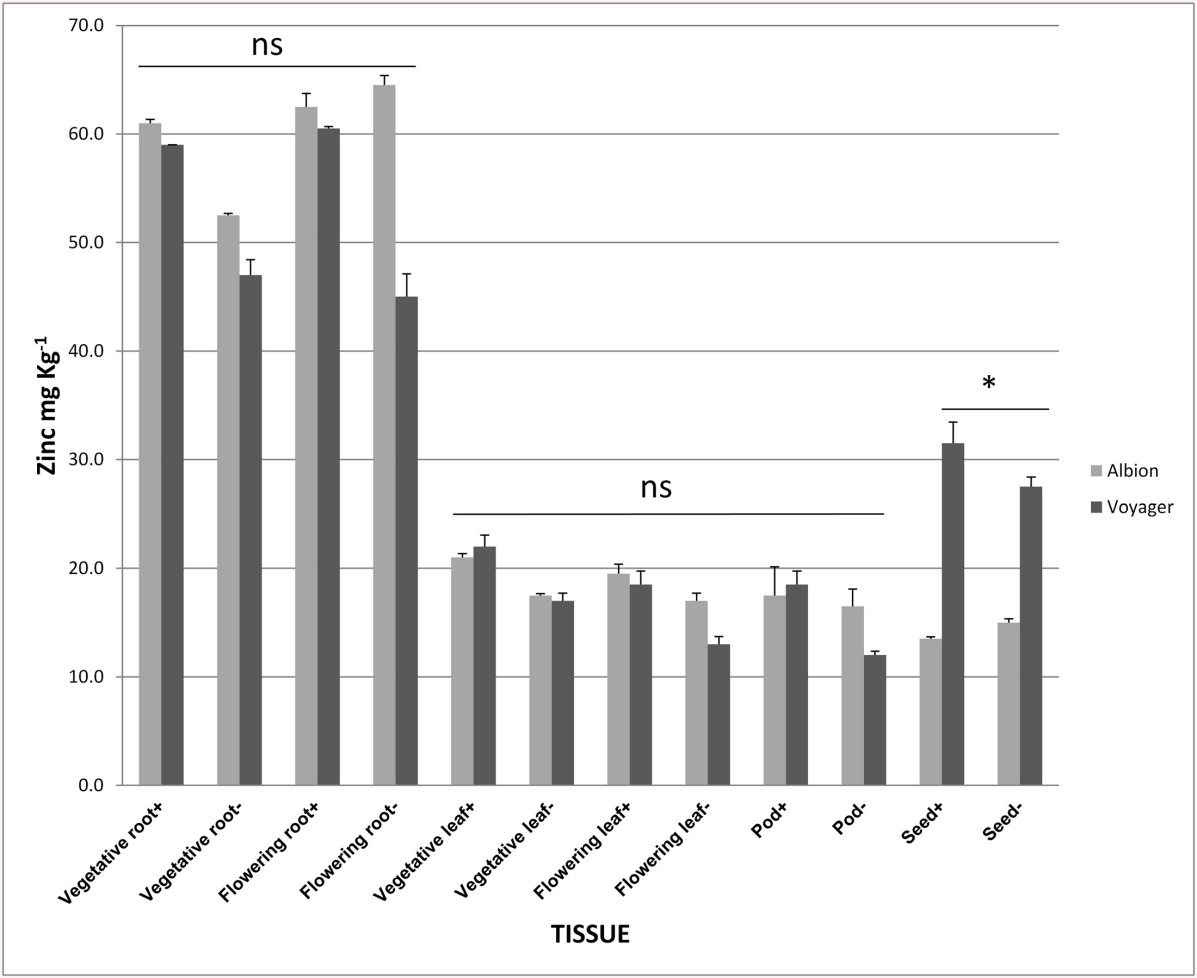
Zinc concentration of roots, leaves, pods and seed of two bean genotypes grown under normal Zn and no Zn fertilization.

Voyager and Albion are two navy bean cultivars. Voyager has been shown to contain higher seed Zn than Albion and these differences have been noted in both field and controlled environment experiments [25,26]. In order to determine if the difference in Zn levels between Voyager and Albion is limited to the seed, Zn levels were measured in roots, leaves, pods, and seeds of each genotype under two Zn fertilization treatments. Plants grown under the low Zn fertilization treatment had reduced Zn levels in the roots and leaves during vegetative growth, leaves during flowering, and pods for both genotypes. Zinc levels between Albion and Voyager were not significantly different for each tissue evaluated, except for seeds, where Voyager had 1.8 to 2.3 fold higher seed Zn levels (Fig 1). Zn content on a per seed basis was also calculated to determine if the seed Zn differences between the genotypes were due to seed size differences. On a per seed basis, Voyager had 3 times and 1.6 times more Zn than Albion under normal and no Zn fertilization respectively (data not shown). The higher seed Zn content in Voyager indicate that the differences in seed Zn concentration observed are not due to a dilution effect because of seed size. The importance of seed size in influencing seed micronutrient concentrations has been observed in genetic studies with *Medicago truncatula* and *P*. *vulgaris* [36,37]. Previous physiological and fertilization studies with Albion and Voyager showed that Albion accumulated higher Zn levels in stems, leaves and pod walls than Voyager. Voyager also had higher seed yield than Albion under low Zn fertilization and similar seed yield under normal and high Zn fertilization treatments [26]. Based on these findings, it appears that Voyager is better able to remobilize Zn within the plant and transport Zn to seeds. Since very little is known about genes involved in Zn transport into developing seeds, and it appears that root and leaf Zn levels are similar between the two genotypes, we decided to study the transcriptome of developing pods for potential clues on genes involved in Zn remobilization to the seeds. The seed Zn concentration differences between Voyager and Albion have been shown to be controlled by a single gene [25] thereby making these two genotypes excellent candidates for transcriptome analysis to identify genes responsible for the seed Zn differences.

### Pod Transcriptome Characterization

Voyager and Albion were grown in a replicated greenhouse experiment. Under these growing conditions Voyager had 1.4 times more seed Zn than Albion (Table 1). These values observed in greenhouse grown plants are similar to what has been observed for these genotypes in some field studies in Michigan (Cichy, unpublished). Seed Fe levels were variable in the greenhouse grown plants and therefore no significant differences were detected. However other studies have shown Voyager to have higher seed Fe than Albion [38]. Voyager also had 12.5% more N in the seed than Albion (Table 1). Positive correlations between seed Zn and N have been found in a number of crops including wheat and beans [39,40].

**Table 1:** Mean concentration of zinc, iron, and nitrogen in pods and seeds of Albion and Voyager plants from which RNA samples for sequencing were taken.

	Pod		Seed		
	Zn (ug g^-1^)	Fe (ug g^-1^)	Zn (ug g^-1^)	Fe (ug g^-1^)	N (%)
**Voyager**	36^a^	92^a^	42^a^	101^a^	3.19^a^
**Albion**	28^b^	75^b^	29^b^	88^a^	2.79^b^

Means followed by the same letter in a column are not significantly different at P = 0.05

Developing pods were collected from the greenhouse grown plants at 12 days after flowering. At this developmental stage, 84% of pod weight was the pod wall and 15% was the developing seed in Voyager and 66% was the pod wall and 34% was the developing seed in Albion. This developmental stage has been characterized as the time prior to seed filling and when nitrogen is accumulating in the pods [41].

**Fig 2:**
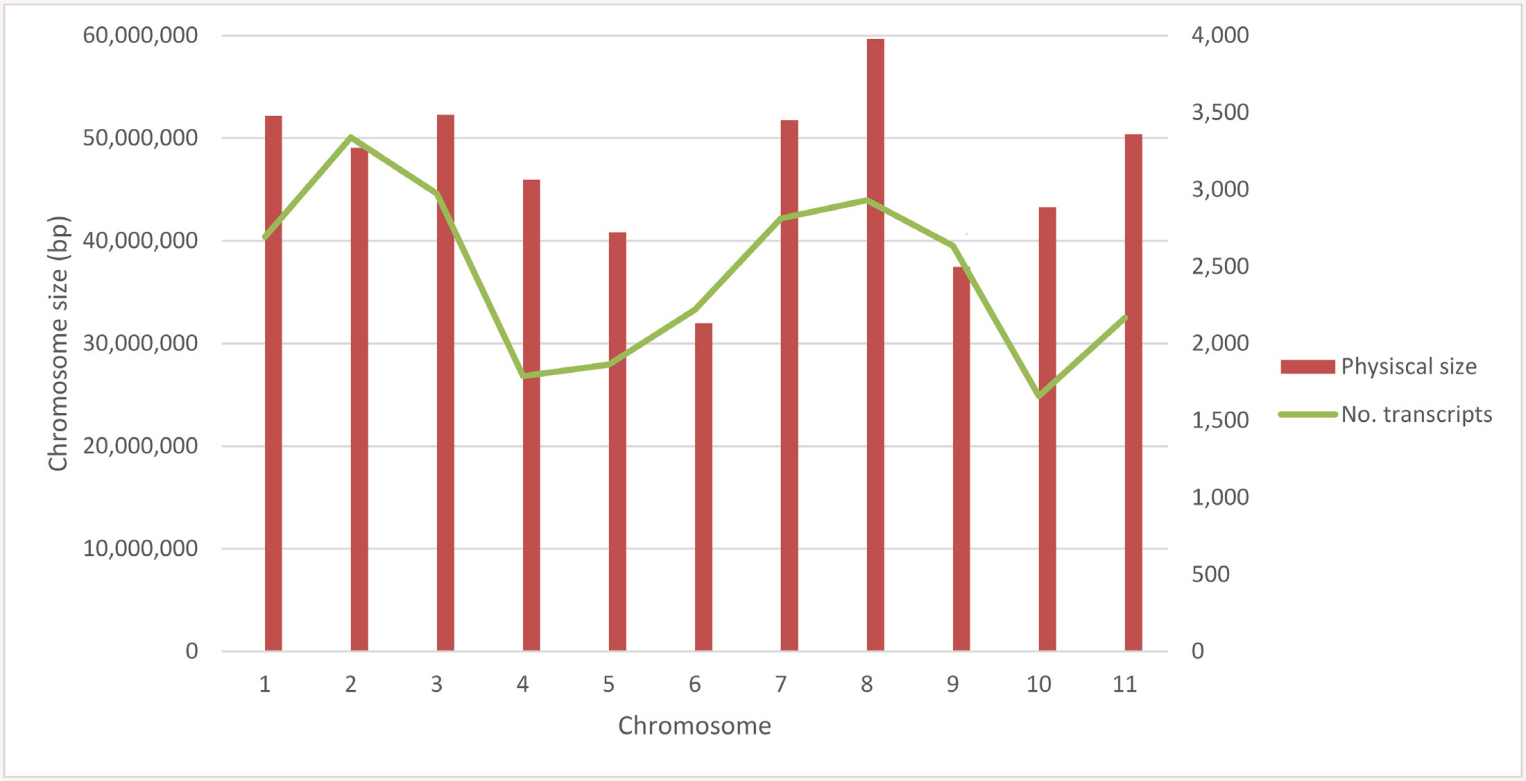
Number of transcripts and their distribution along of eleven chromosomes of common bean.

Voyager and Albion pod RNA was sequenced as 75 bp paired end reads. RNA reads were mapped to the *P*. *vulgaris* genome sequence, v 1.0 DOE-JGI and USDA-NIFA http://www.phytozome.net) [28] with an average of 31,505,836 high quality mapped reads per sample. A total of 27,197 genes were detected in the transcriptome of developing pods. Analysis of the number of transcripts per chromosome showed that chromosomes 1, 2, 3, 7, 8, and 9 contained the highest number of transcripts. Chromosome 10 had the lowest number of transcripts. In dry bean mapping populations, few quantitative trait loci have been found on chromosome 10 and it is often difficult to identify polymorphic markers on this chromosome, indicating a low rate of recombination [42]. A positive correlation (r = 0.5; P<0.05) between size of the chromosome and number of transcript was discovered. Chromosomes 2, 3, 7, and 11 had the highest number of highly expressed genes in the pods (Fig 2).

**Fig 3:**
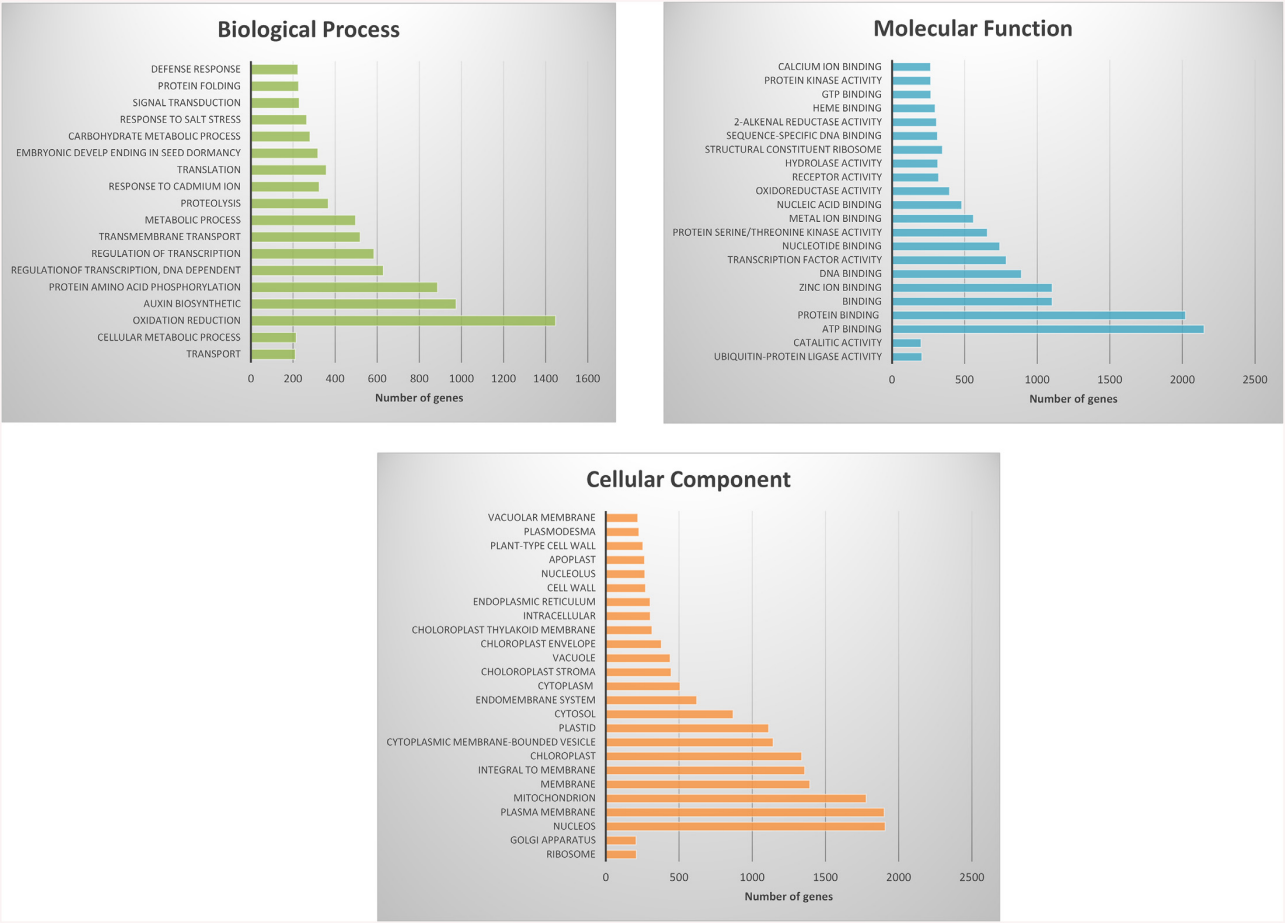
Histogram representation of GO classification. Transcripts of developing pods were annotated in three categories: biological processes (3a), molecular function (3b) and cellular components (3c).

**Fig 4:**
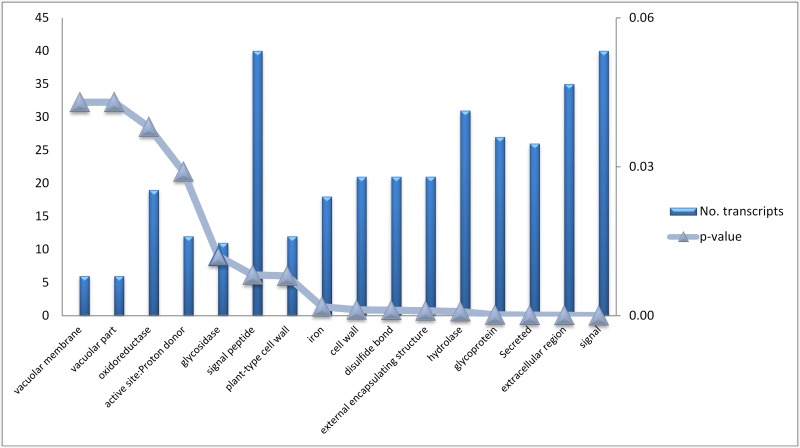
Enrichment analysis of 381 differential expressed genes between Albion and Voyager. X axis indicates GO terms where genes were grouped, left Y axes indicate the number of genes and the right Y axis indicates *p-values* show significance of association of genes with GO term.

Gene annotation was achieved using Blast2Go (using BLASTx and E-value ^-6^ as parameters). Using gene ontology, graphs were developed which classify gene expression in the bean pods. Transcripts expressed during this pod developmental stage were most related to oxidation reduction, auxin biosynthesis and amino acid phosphorylation (Fig 3a). Transcripts functioning in the nucleus and plasma membrane were the most represented cell types in the developing bean pods (Fig 3b). Transcripts related to ATP binding and protein binding were the most highly abundant gene types (Fig 3c). Enrichment analysis of differential expression genes determined their association to a specify pathway [31]. Genes were clustered into nine GO categories according to the David bioinformatics database as shown in Fig 4.

Genes highly expressed in developing pods included several seed maturation proteins, acid phosphatase and lipid synthesis related genes. The most highly expressed genes in the transcriptome of developing pods of dry beans were haloacid dehalogenase IIIB acid phosphatase (HAD), lipid transfer protein 3 (LTP3), PAP85 cupin protein, PRXR1 peroxidase protein, and phaseolin. The HAD IIIB acid phosphatase belongs to a family of plant phosphatases. Some members of this family have been annotated as vegetative storage proteins (VSP) highly expressed in soybean leaves and also abundant in *A*. *thaliana* flowers [43]. LTP3 (lipid transfer protein 3) transfer several different phospholipids and can bind fatty acids and could play a major role in membrane biogenesis [44]. PAP85 cupin family are metalloenzymes with two motif conserved sequences which act as ligands for the binding of an active-site metal ion, such as Fe, Mn, or Zn [45]. Additionally, they encode seed storage proteins and are involved in the regulation of nitrogen utilization [46]. Phaseolin is the seed storage protein most abundant in *P*. *vulgaris* seeds [47]. It is a glycoprotein formed by two genes, the α and β phaseolin genes with a relative electrophoretic diversity useful for discriminating geographical origin and wild and domesticated beans [47,48]. These results demonstrated that in *P*. *vulgaris*, storage protein transcripts were the most abundant process beginning at R5 and R6 growth stage which involve storage product accumulation, phases of cell expansion and synthesis of reserve metabolites [49].

### Differential Expression

The expression levels of genes in developing pods were measured in fragments per kilobase of exon model per million mapped reads (FPKM). With this criterion,19,510 expressed genes were detected in Albion and 19,527 expressed genes in Voyager. We determined the correlation between normalized sequencing counts and RT qPCR gene expression data and there was a high correlation between the two methodologies (r = 0.85 p = 0.05) (Table A in S1 File).

The distribution of gene expression values in log_10_ was left-skewed; the median and mean FPKM values are 11.11 and 45.43 respectively. There were 381 differentially expressed genes in Albion and Voyager of which 6 were exclusively expressed in Albion and 3 genes were only expressed in Voyager. Genes with the highest differential expression patterns between the two genotypes included cysteine proteinases and MLP-like protein-43 more highly expressed in Albion pods (Table 2). These genes are related to growth and mobilization and accumulation of storage proteins in seeds in development [50]. In Voyager, the genes most highly differentially expressed as compared to Albion were cinnamoyl-CoA reductase (CCR-like) and 2Fe-2S ferredoxin-like. Both of these genes are related to metal ion transport in addition to abscisic acid biosynthesis and the electron transport chain. Differential expression analysis showed that the cinnamoyl-CoA reductase (CCR-like) and 2Fe-2S ferredoxin-like genes were 1.65 and 1.42 respectively more expressed in Voyager pods than Albion pods.

**Table 2:** Genes and function of the most highly differential expressed in Albion and Voyager.

Genotype	Function	Albion^a^	Voyager^a^	log2 (Fold Change)^b^ ^,^ ^c^
**Albion**				
Cysteine proteinases	Extracellular proteinase probably having a crucial role during rapid cell growth and leaf expansion	791	203	-2
MLP-like protein 43	Associated with fruit and flower development and pathogen defense responses	732	98	-3
SCR-like 11	S locus cysteine-rich protein	392	66	-3
Serine carboxypeptidase-like	Serine-type carboxypeptidase activity involved in proteolysis	237	81	-2
Aspartic proteinase A1	Encodes an aspartic proteinase that forms a heterodimer and is stable over a broad pH range	231	76	-2
Low-molecular-weight cysteine-rich	Predicted to encode a PR (pathogenesis-related) protein.	215	31	-3
**Voyager**				
CCR-like	Cellular cation homeostasis, divalent metal ion transport. Expressed in embryo axis, cotyledons.	146	460	2
2Fe-2S ferredoxin	Abscisic acid biosynthetic process, electron transport chain, pentose-phosphate shunt.	110	295	1
Zinc-binding ribosomal protein	DNA recognition, RNA packaging, transcriptional activation, regulation of apoptosis, protein folding and assembly, and lipid binding.	74	169	1
Adenine nucleotide alpha hydrolases	Function unknown. Involved in response to stress. Expressed during petal differentiation and expansion stage.	65	162	1
Bifunctional inhibitor/lipid-transfer protein/seed storage 2S albumin superfamily protein	Function in lipid binding. Located in endomembrane system. Expressed in shoot apex, embryo, flower, leaf, seed. Expressed during cotyledon expansion stage.	35	161	2
Basic chitinase	Defense response after wounding or pathogenic attack	10	150	4

^a^ The value used to compute significance of the observed change of transcript abundance was measured in fragments per kilobase of transcript per million mapped reads (FPKM.)

^b^ Log2 (Fold change): negative values indicate that the gene is more highly expressed in Albion and positive values indicate the gene is more highly expressed in Voyager.

^c^ All genes were significantly differential expressed FDR<0.05

**Fig 5:**
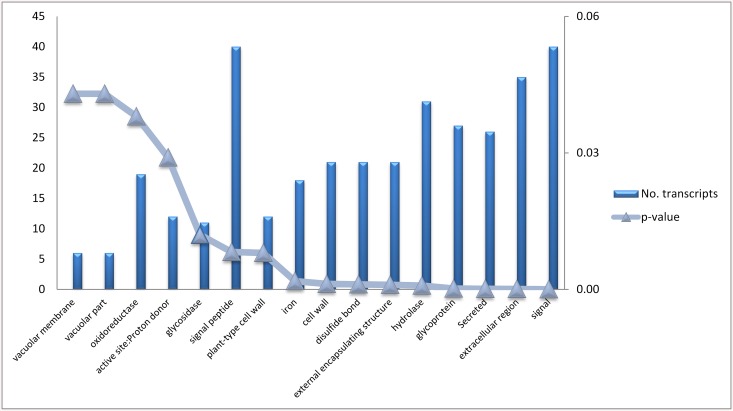
Expression levels of the ten gene families involved in Zn transport and accumulation identified in developing pod transcriptome. Bars represent each gene family and colors correspond to different member within each family. Height of each bar represents gene fold change in Albion and Voyager. ZIP (zinc-regulated transporter, iron regulated transporter like), NRAMP (natural resistance associated macrophage protein), bZIP (bZip transcription factor), ZIF (zinc-induced facilitator), HMA (heavy metal associated), VIT (vacuolar iron transport), YSL (yellow stripe like), NAS (nicotiamine synthase), FRO (Iron reductase) and ferritin.

Gene families known to be involved in Zn transport were identified and their expression in the developing pods of Albion and Voyager was quantified (Fig 5). These families included ZRT and IRT–like protein (ZIP), basic region/leucine zipper motif (bZIP) transcription factors, vacuolar iron transport (VIT), natural resistance-associated macrophage protein (NRAMP), zinc induced facilitator (ZIF), yellow stripe (YSL), heavy metal ATPase (HMA), nicotianamine synthase (NAS), dehydrin, and metallothionein. The ZRT and IRT–like protein (ZIP) family is involved in uptake, transport to leaves and translocation to seeds, embryo, endosperm, and seed coat of zinc [51]. It was the largest family and 20 out of 23 members [34] were found in the developing pod transcriptome of which fifteen were expressed (Table 3). Expression analysis of this family in dry bean showed that some members were highly expressed in leaves and pods under two Zn treatments [34]. Additionally, in *Arabidopsis* and maize, some members of this family are preferentially expressed in the embryo and endosperm [52]. These results emphasize the importance of these genes in Zn transport into sink organs. bZIP transcription factors bZIP19 and bZIP23 in *Arabidopsis*, were associated to promoter regions of the zinc deficiency-induced ZIP4 gene of ZIP family [9]. These bZIPs belong to group F, have been described containing two DNA binding domains needed to respond to low zinc supply in *Arabidopsis* [9]. Two bZIP basic leucine-zipper transcription factor genes homologous to bZIP23 in *Arabidopsis* were identified and both were expressed in pods.

**Table 3:** Gene families involved in Zn and/or Fe transport and expression analysis in the developing pods of Albion and Voyager.

Gene family	Homologous *A*. *thaliana*	Chromosome	Position	Albion^a^	Voyager^a^	fold change^b^	FDR^c^
PvbZIP1	bZIP23 basic-leucine zipper	Chr05	3,212,438	22	23	0.0	
PvbZIP2	bZIP23 basic-leucine zipper	Chr11	3,134,439	41	46	0.2	
PvNRAM1	ATNRAMP, metal ion transporter	Chr01	44,116,444	26	31	0.3	
PvNRAM2	ATNRAMP3, metal ion transporter	Chr02	1,609,575	61	62	0.0	
PvNRAM3	ATNRAMP3, metal ion transporter	Chr03	46,129,963	12	11	-0.2	
PvNRAM4	ATNRAMP6, metal ion transporter 6	Chr05	40,351,734	17	17	0.0	
PvNRAM5	ATNRAMP, metal ion transporter	Chr07	37,134,084	30	34	0.2	
PvNRAM6	ATNRAMP2, metal ion transporter 2	Chr09	11,751,007	19	23	0.3	
PvNRAM7	ATNRAMP6, metal ion transporter 6	Chr09	18,914,511	14	12	-0.3	
PvNRAM8	ATNRAMP2, metal ion transporter 2	Chr10	37,315,780	0	0	0.0	
PvNRAM9	ATNRAMP6, metal ion transporter 6	Chr10	42,893,083	2	0	-2.3	*
PvZIF1	ZIFL2, zinc induced facilitator-like 2	Chr02	21,890,013	1	19	4.6	*
PvZIF2	ZIFL1, zinc induced facilitator-like 1	Chr05	1,050,386	3	6	0.9	
PvZIF3	ZIFL1, zinc induced facilitator-like 1	Chr11	44,602,386	0	0	0.0	
PvZIF4	ZIFL1, zinc induced facilitator-like 1	Chr11	44,656,239	0	0	0.0	
PvZIF5	ZIFL1, zinc induced facilitator-like 1	Chr11	44,662,432	0	0	-0.4	
PvZIF6	ZIFL1, zinc induced facilitator-like 1	Chr11	46,613,123	9	11	0.2	
PvZIF7	ZIFL1, zinc induced facilitator-like 1	Chr11	46,625,565	0	0	0.0	
PvZIF8	ZIFL1, zinc induced facilitator-like 1	Chr11	46,638,452	0	0	0.0	
PvZIF9	ZIFL1, zinc induced facilitator-like 1	Chr11	46,652,668	4	3	-0.4	
PvZIF10	ZIFL1, zinc induced facilitator-like 1	Chr11	46,667,766	18	8	-1.1	
PvZIP1	ATZIP4, zinc transporter 4 precursor	Chr01	3,438,922	3	3	0.1	
PvZIP2	ZIP10, zinc transporter 10 precursor	Chr02	19,642,778	0	0	0.1	
PvZIP3	ZIP metal ion transporter family	Chr02	33,721,809	19	20	0.0	
PvZIP4	ZIP10, zinc transporter 10 precursor	Chr03	49,001,484	0	0	0.0	
PvZIP5	ZIP10, zinc transporter 10 precursor	Chr03	49,013,792	0	0	0.0	
PvZIP6	ZIP11, zinc transporter 11 precursor	Chr05	37,425,474	0	0	0.0	
PvZIP7	ZIP11, zinc transporter 11 precursor	Chr05	37,429,894	1	2	1.2	
PvZIP8	ZIP1, zinc transporter 1 precursor	Chr05	5,642,976	6	8	0.4	
PvZIP9	ZTP29, ZIP metal ion transporter family	Chr05	37,714,954	18	17	-0.1	
PvZIP10	ZIP11, zinc transporter 11 precursor	Chr06	17,173,381	12	21	0.8	
PvZIP11	ZIP1, zinc transporter 1 precursor	Chr06	199,508	1	1	-0.2	
PvZIP12	ZIP5, zinc transporter 5 precursor	Chr06	1,040,877	2	8	2.2	*
PvZIP13	ATZIP6, metal ion transporter family	Chr06	18,953,200	7	8	0.2	
PvZIP14	ZIP metal ion transporter family	Chr08	7,633,778	11	12	0.1	
PvZIP15	ZIP5, zinc transporter 5 precursor	Chr08	59,348,008	8	15	0.9	
PvZIP16	ATZIP6, metal ion transporter family	Chr08	57,181,379	21	24	0.2	
PvZIP17	ATIRT3, iron regulated transporter 3	Chr09	12,668,955	31	36	0.0	
PvZIP18	ZIP metal ion transporter family	Chr10	9,814,851	2	2	0.1	
PvZIP19	ZTP29, ZIP metal ion transporter family	Chr11	5,068,287	1	1	0.0	
PvZIP20	ZIP10, zinc transporter 10 precursor	scaff	1,071	0	1	0.2	
PvVIT1	ATVIT1, vacuolar iron transporter 1	Chr02	48,170,585	2	1	-0.5	
PvVIT2	ATVIT1, vacuolar iron transporter 1	Chr02	48,175,491	14	18	0.4	
PvVIT3	ATVIT1, vacuolar iron transporter 1	Chr02	48,252,436	12	14	0.2	
PvVIT4	Vacuolar iron transporter (VIT)	Chr02	23,134,245	0	0	0.0	
PvVIT5	Vacuolar iron transporter (VIT)	Chr02	36,507,752	0	0	-1.5	
PvVIT6	Vacuolar iron transporter (VIT)	Chr02	36,521,460	0	0	-0.8	
PvVIT7	Vacuolar iron transporter (VIT)	Chr02	36,533,751	0	0	0.4	
PvVIT8	Vacuolar iron transporter (VIT)	Chr02	36,541,077	0	0	0.0	
PvVIT9	Vacuolar iron transporter (VIT)	Chr04	27,416,262	10	19	0.9	
PvVIT10	Vacuolar iron transporter (VIT)	Chr07	7,508,398	28	38	0.4	
PvVIT11	ATVIT1, vacuolar iron transporter 1	Chr08	6,284,802	6	7	0.4	
PvVIT12	Vacuolar iron transporter (VIT)	Chr08	49,131,247	6	6	0.0	
PvVIT13	vacuolar iron transporter (VIT)	Chr09	8,164,280	8	7	0.0	
PvVIT14	Vacuolar iron transporter (VIT)	Chr10	3,221,741	1	1	0.1	
PvVIT15	vacuolar iron transporter (VIT)	Chr10	3,229,195	0	0	1.0	
PvYSL1	ATYSL1, Yellow stripe like 1	Chr01	13,421,083	18	28	0.0	
PvYSL2	YSL6, Yellow stripe like 6	Chr01	16,152,062	60	65	0.1	
PvYSL3	YSL7, Yellow stripe like 7	Chr03	626,298	16	18	0.1	
PvYSL4	YSL7, Yellow stripe like 7	Chr03	631,299	2	2	0.0	
PvYSL5	ATYSL1, Yellow stripe like 1	Chr04	21,588,269	3	4	0.4	
PvYSL6	YSL7, Yellow stripe like 7	Chr04	41,773,229	17	15	-0.1	
PvYSL7	YSL7, Yellow stripe like 7	Chr06	20,249,225	0	0	0.0	
PvYSL8	ATYSL3, Yellow stripe like 3	Chr08	40,137,873	4	9	0.0	
PvYSL9	ATYSL3, Yellow stripe like 3	Chr09	9,292,230	62	72	0.0	
PvHMA1	HMA5, heavy metal atpase 5	Chr02	29,860,709	11	5	-1.1	
PvHMA2	HMA5, heavy metal atpase 5	Chr02	29,878,666	1	0	-0.6	
PvHMA3	HMA5, heavy metal atpase 5	Chr02	45,175,820	0	0	0.0	
PvHMA4	HMA5, heavy metal atpase 5	Chr02	45,187,687	0	0	0.3	
PvHMA5	HMA6, PAA1P-type ATP-ase 1	Chr02	36,870,747	5	6	0.3	
PvHMA6	HMA7, copper-transporting ATPase	Chr02	34,600,085	13	13	0.0	
PvHMA7	ATHMA1, heavy metal atpase 1	Chr03	5,628,284	30	26	-0.2	
PvHMA8	ATHMA1, heavy metal atpase 1	Chr03	46,285,474	26	25	-0.1	
PvHMA9	ATHMA2, heavy metal atpase 2	Chr03	33,726,455	20	19	-0.1	
PvHMA10	ATHMA4, heavy metal atpase 4	Chr09	35,288,969	1	1	-0.5	
PvHMA11	ATHMA8, type ATPase	Chr09	13,120,413	8	7	-0.2	
PvHMA12	HMA7, copper-transporting ATPase	Chr09	35,544,425	33	35	0.1	
PvHMA13	HMA5, heavy metal atpase 5	Chr10	3,512,059	3	3	0.1	
PvNAS1	ATNAS2, nicotianamine synthase 2	Chr01	48,680,147	0	0	-0.2	
PvNAS2	ATNAS4, nicotianamine synthase 4	Chr05	6,792,803	0	0	0.0	
PvNAS3	ATNAS4, nicotianamine synthase 4	Chr06	23,217,021	0	0	-0.1	
PvDehydrin	Dehydrin	Chr04	44,048,043	2	1	-1.8	
PvDehydrin	Dehydrin	Chr09	921,414	24	14	-0.8	
PvDehydrin	Dehydrin	Chr11	49,334,383	0	0	0.3	
PvMT	metallothionein 2A	Chr08	11,131,986	3262	3989	0.3	
PvMT	metallothionein 2A	Chr10	1,509,086	669	847	0.3	
PvMT	metallothionein 2A	Chr10	1,905,781	310	246	-0.3	
PvMT	metallothionein 2A	Chr10	1,900,737	0	1	1.1	

^a^ The value used to compute significance of the observed change of transcript abundance was measured in fragments per kilobase of transcript per million mapped reads (FPKM)

^b^ Log2 (Fold change): negative values indicate that the gene is more highly expressed in Albion and positive values indicate the gene is more highly expressed in Voyager

^c^ *indicates genes were significantly differential expressed FDR<0.05

We identified fifteen vacuolar iron transport (VIT) genes which nine members were expressed in developing pods. Relatively low levels of expression (0 to 38 FPKM) was observed. In *Arabidopsis*, VIT1 has been found highly expressed in the developing seeds and mediates iron storage in the embryo [53]. Nicotianamine synthase (NAS) is a metal chelator that produces a nonproteinogenic amino acid which binding a variety of transition metals [54,55]. Metallothionein proteins bind transition metals and play a role in the homeostasis and detoxification of non-essential minerals [56]. Four metallothionein genes were identified in the developing pods and three of them were highly expressed (246 to 3,989 FPKM) but none were differentially expressed. In *Arabidopsis*, AtMT4a and AtMT4b have been suggested to be involved in Zn storage in seeds [57].

Very few of the Zn and/or Fe transport related genes were differentially expressed. Those that were differentially expressed included one gene each from NRAMP, ZIP and ZIF families (Table 3). A member of NRAMP was expressed in Albion and not expressed in Voyager (2.3 fold change). NRAMP family genes have been involved mainly in iron translocation [21,22]. However, complementation analysis showed that a member of NRAMP was able to complement growth in yeast [23]. PvZIF1and PvZIP12 were more expressed in Voyager than Albion in 4.6 and 2.2 fold change respectively. ZIP and ZIF family genes have been shown to be involved in transportation of minerals to the vacuole and transport to seeds in *Arabidopsis* [8,23]. This suggests that one possible reason why Albion has lower seed Zn that Voyager is because it is being moved to the vacuoles and not transported to the seed. However, for each Zn/Fe related family analyzed transcripts were from 1 to 72 of FPKM value which was relatively low as compared to those genes related to lipid synthesis and storage protein. Therefore future studies may need to target earlier and later developmental time points to better characterize genotypic differences in Zn remobilization.

The transcript sequences from Voyager and Albion were also analyzed for SNPs. A total of 12,118 SNPs were identified between the two genotypes (S2 File). In total 3,401 genes contained SNPs and on average there were 3.6 SNPs per gene. Sanger sequencing was used to validate SNPs in nine genes (Table 4). Of the gene families related to Zn and/or Fe transport, eleven genes were found to have SNPs with a total of 47 SNPs average of four SNPs per gene. In total, 15 of the SNPs resulted in an amino acid change (Table 5). Of the genes with SNPs, the same ZIF gene (Phvul.002G108300) which was more highly expressed in Albion than Voyager contained 10 SNPs in the coding region. This gene maps to chromosome 11 and while numerous studies have reported QTL for seed Fe and Zn concentration [37,58,59], this ZIF gene is not within those regions. It is interesting to note that the HMA gene (Phvul002G288300 and Phvul002G19000) maps to chromosome 2 in a region where a major QTL for seed Zn concentration has been identified in bean RIL populations from both Mesoamerican and Andean intra gene pool crosses [37,59]. The seed Zn differences in Albion and Voyager with single marker genetic analysis indicated that this trait associated with SSR markers BM154 and BM184 found on chromosome 9 [38]. Based on the physical position of these markers on chromosome 9 (1,856,660 1,718,891bp respectively) genes such as dehydrin and bZIP44 were found in the surrounding region (922,386 and 1,006,283). Dehydrins are responsible for osmotic stress from drought, cold, and high salinity but also binds metals reducing metal toxicity in plant cells under water-stressed conditions [60].

**Table 4:** Forward and reverse sequence for all primer pairs used to validate putative SNPs in genotypes Albion and Voyager.

*P*. *vulgaris* genome ID	Gene	Forward primer	Reverse primer	Product Size (bp)
Phvul.008G157800	PvYSL	CGCTATGTCGTAACACTTCTGCACC	TTTGTGCTTGCTGCCTTAGGTGGG	741
	PvYSL	GGGAAGGGCAGAAAAGCCTTCGAC	TTGCCTTGATCCTCGGTGATGGGT	874
Phvul002G19000	PvHMA	AACCTCTCACCGCGACCTCACTAC	CCCACACAACAACCCCATCGGAAG	1483
	PvHMA	AACCTCTCACCGCGACCTCACTAC	CATGTTCGCAGATCCACGGCGTAA	1131
	PvHMA	GACACGGCGGTTTTGCTGACTTTG	GCACTCTCTAATGCCTGCCCTCCT	746
	PvHMA	GTTGGTGCATCTCAGGGTGTGCTC	GGCCAATGGATGCTCACTATTCACCT	853
Phvul.011G173100	PvZIF	GCCCAGCATTGGGAGGCTATTTGG	AAGCCACATTCGGAACATGACCGC	1211
	PvZIF	CGTGACGTGTGCAATGATGCCACT	CGCACCAACAACACAAAACAGGGA	561
	PvZIF	AGGTGGTGCAGTGTGAGTGTTCCT	AGGCAAGTTACAGATTGAATTGGTTCCCT	886

**Table 5:** Identification of SNPs in genes that are members of Zn and/or Fe transport-related families, followed by the length of the CDS, genomic length, number of SNPs between Albion and Voyager, whether those SNPs validated via PCR and if the SNPs resulted in an amino acid change.

P. vulgaris genome ID	Family	CDS Length	Genomic Length	SNPs in CDS	Chr	SNPs confirmed^a^	AA change
Phvul002G288300	HMA	2,982	5,648	5	2		Syn, Syn, Syn, Syn, Phe/Ser
Phvul002G19000	HMA	2,958	4,150	6	2	*	Syn, Syn, Syn, Syn, Syn, Syn
Phvul.002G208800	HMA	2,835	24,712	1	2		Ala/Thr
Phvul.003G047300	HMA	3,564	9,174	4	3		Syn, Syn, Syn, Syn
Phvul.002G014300	NRAMP	1,524	2,952	4	2		Val/Leu, Syn, Syn, Syn
Phvul.008G157800	YSL	1,908	2,751	3	8	*	Met/Ile, Syn, Syn
Phvul.001G081600	YSL	2,031	6,538	2	1		Gly/Ser, Syn
Phvul.011G173100	ZIF	1,470	5,330	10	11	*	Gln/Leu, Asp/Glu, Arg/Pro, Ala/Val, Syn, Syn, Syn, Thr/Ile, Val/Ile, Gln/His
Phvul.011G189500	ZIF	1,470	4,785	5	11		Syn, Ile/Val, Syn, Syn, Syn
Phvul.008G221200	Ferritin	891	2,550	1	8		Glu/Lys

^a^: * indicates SNPs were confirmed by PCR amplification and sequencing.

Accumulation of minerals in the seed involved several complex and still unknown mechanisms. The ability to uptake and accumulate minerals, how much mineral is absorbed by roots, transfer into the shoots and leaves via the xylem, and translocation to seeds via the phloem are all potentially important genetic regulation points. It still unclear which mechanism is the most important step in terms of uptake, transport, remobilization and accumulation to determine where our effort to increase concentration of Zn in seed should focus. In *Pisum sativum*, Zn remobilization from vegetative tissues to the seeds has been measured and 75–95% of mineral content in pods was remobilized to the seed tissue [61]. In this study we reported the main genes that likely are related to Zn remobilization during the seed filling period.

RNA sequencing was used to identify members of mineral transporter gene families expressed during bean pod development. The comparative analysis of two closely related bean genotypes with different levels of seed Zn indicate which genes are differentially expressed and which contain SNPs. This information is useful to identify candidate genes for seed mineral biofortification and the most promising candidate from this study is the ZIF gene (Phvul.002G108300).

## Supporting Information

S1 File This file contains the description of validation of the RNA-seq results with quantitative RT-PC and supplementary tables. **Table A**. Comparison of fold change of PvZIP and Pv bZIP obtained from RNA-seq and qRT PCR validation of PvZIP and Pv bZIP genes in developing pods. Correlation between normalized sequencing counts in RNA-seq and expression determined by RT qPCR showed high correlation between two methodologies (r = 0.85 p = 0.05). **Table B.** Primer list for gene expression analysis via RT-qPCR. Four member of the ZIP gene family and two member of the bZIP family were amplified via RT-qPCR to validate the expression profiles obtained by RNA-seq.
(DOCX)

S2 File List of SNPs found in the pod transcriptome of Albion and Voyager. (XLSX)

## References

[1] Chasapis CT, Loutsidou AC, Spiliopoulou CA, Stefanidou ME (2012) Zinc and human health: an update. Archives of Toxicology 86: 521–534. 10.1007/s00204-011-0775-1 22071549

[2] Hambidge M (2000) Human zinc deficiency. The Journal of Nutrition 130: 1344S–1349S. 10801941 10.1093/jn/130.5.1344S

[3] Sandstead HH (1991) Zinc deficiency: a public health problem? American Journal of Diseases of Children 145: 853–859. 1858720 10.1001/archpedi.1991.02160080029016

[4] Bouis HE, Hotz C, McClafferty B, Meenakshi J, Pfeiffer WH (2011) Biofortification: a new tool to reduce micronutrient malnutrition. Food & Nutrition Bulletin 32: 31S–40S. 10.1177/15648265110321S105 21717916

[5] Islam F, Basford K, Jara C, Redden R, Beebe S (2002) Seed compositional and disease resistance differences among gene pools in cultivated common bean. Genetic Resources and Crop Evolution 49: 285–293.

[6] Blair MW, González LF, Kimani PM, Butare L (2010) Genetic diversity, inter-gene pool introgression and nutritional quality of common beans (Phaseolus vulgaris L.) from Central Africa. Theoretical and Applied Genetics 121: 237–248. 10.1007/s00122-010-1305-x 20224891 PMC2886139

[7] Waters BM, Sankaran RP (2011) Moving micronutrients from the soil to the seeds: genes and physiological processes from a biofortification perspective. Plant Science 180: 562–574. 10.1016/j.plantsci.2010.12.003 21421405

[8] Guerinot ML (2000) The ZIP family of metal transporters. Biochimica et Biophysica Acta (BBA)-Biomembranes 1465: 190–198. 10748254 10.1016/s0005-2736(00)00138-3

[9] Assunção AG, Herrero E, Lin Y-F, Huettel B, Talukdar S, Smaczniak C, et al (2010) Arabidopsis thaliana transcription factors bZIP19 and bZIP23 regulate the adaptation to zinc deficiency. Proceedings of the National Academy of Sciences: 201004788. 10.1073/pnas.1004788107 PMC2890486 20479230

[10] Waters BM, Chu H-H, DiDonato RJ, Roberts LA, Eisley RB, Lahner B, et al (2006) Mutations in Arabidopsis yellow stripe-like1 and yellow stripe-like3 reveal their roles in metal ion homeostasis and loading of metal ions in seeds. Plant Physiology 141: 1446–1458. 16815956 10.1104/pp.106.082586 PMC1533956

[11] Curie C, Cassin G, Couch D, Divol F, Higuchi K, Le Jean M, et al (2009) Metal movement within the plant: contribution of nicotianamine and yellow stripe 1-like transporters. Annals of Botany 103: 1–11. 10.1093/aob/mcn207 18977764 PMC2707284

[12] Haydon MJ, Kawachi M, Wirtz M, Hillmer S, Hell R, Kramer U (2012) Vacuolar nicotianamine has critical and distinct roles under iron deficiency and for zinc sequestration in Arabidopsis. The Plant Cell Online 24: 724–737. 10.1105/tpc.111.095042 PMC3315243 22374397

[13] Schuler M, Rellán-Álvarez R, Fink-Straube C, Abadía J, Bauer P (2012) Nicotianamine functions in the phloem-based transport of iron to sink organs, in pollen development and pollen tube growth in Arabidopsis. The Plant Cell Online 24: 2380–2400. 10.1105/tpc.112.099077 PMC3406910 22706286

[14] Samardjieva KA, Tavares F, Pissarra J (2015) Histological and ultrastructural evidence for zinc sequestration in *Solanum nigrum* L. Protoplasma 252: 345–357. 10.1007/s00709-014-0683-3 25119835

[15] Morel M, Crouzet J, Gravot A, Auroy P, Leonhardt N, Vavasseur A, et al (2009) AtHMA3, a P1B-ATPase allowing Cd/Zn/Co/Pb vacuolar storage in Arabidopsis. Plant Physiology 149: 894–904. 10.1104/pp.108.130294 19036834 PMC2633814

[16] Haydon MJ, Cobbett CS (2007) A novel major facilitator superfamily protein at the tonoplast influences zinc tolerance and accumulation in Arabidopsis. Plant Physiology 143: 1705–1719. 17277087 10.1104/pp.106.092015 PMC1851824

[17] Ricachenevsky FK, Sperotto RA, Menguer PK, Sperb ER, Lopes KL, Fett JP (2011) ZINC-INDUCED FACILITATOR-LIKE family in plants: lineage-specific expansion in monocotyledons and conserved genomic and expression features among rice (Oryza sativa) paralogs. BMC Plant Biology 11: 20 10.1186/1471-2229-11-20 21266036 PMC3041735

[18] Hanikenne M, Talke IN, Haydon MJ, Lanz C, Nolte A, Motte P, et al (2008) Evolution of metal hyperaccumulation required cis-regulatory changes and triplication of HMA4. Nature 453: 391–395. 10.1038/nature06877 18425111

[19] Ricachenevsky FK, Menguer PK, Sperotto RA, Williams LE, Fett JP (2013) Roles of plant metal tolerance proteins (MTP) in metal storage and potential use in biofortification strategies. Frontiers in Plant Sciences 4. 10.3389/fpls.2013.00144 PMC3653063 23717323

[20] Migocka M, Kosieradzka A, Papierniak A, Maciaszczyk-Dziubinska E, Posyniak E, Garbiec A, et al (2014) Two metal-tolerance proteins, MTP1 and MTP4, are involved in Zn homeostasis and Cd sequestration in cucumber cells. Journal of Experimental Botany: eru 459. 10.1093/jxb/eru459 25422498

[21] Lanquar V, Ramos MS, Lelièvre F, Barbier-Brygoo H, Krieger-Liszkay A, Kramer U, et al (2010) Export of vacuolar manganese by AtNRAMP3 and AtNRAMP4 is required for optimal photosynthesis and growth under manganese deficiency. Plant Physiology 152: 1986–1999. 10.1104/pp.109.150946 20181755 PMC2850043

[22] Thomine S, Lelièvre F, Debarbieux E, Schroeder JI, Barbier-Brygoo H (2003) AtNRAMP3, a multispecific vacuolar metal transporter involved in plant responses to iron deficiency. The Plant Journal 34: 685–695. 12787249 10.1046/j.1365-313x.2003.01760.x

[23] Zhao H, Eide D (1996) The yeast ZRT1 gene encodes the zinc transporter protein of a high-affinity uptake system induced by zinc limitation. Proceedings of the National Academy of Sciences 93: 2454–2458. 10.1073/pnas.93.6.2454 PMC39818 8637895

[24] Olsen LI, Palmgren MG (2014) Many rivers to cross: the journey of zinc from soil to seed. Frontiers in Plant Sciences 5. 10.3389/fpls.2014.00030 PMC3921580 24575104

[25] Cichy KA, Forster S, Grafton KF, Hosfield GL (2005) Inheritance of seed zinc accumulation in navy bean. Crop Science 45: 864–870.

[26] Moraghan JT, Grafton K (1999) Seed-zinc concentration and the zinc-efficiency trait in navy bean. Soil Sci. Soc. Amer. J. 63: 918–922.

[27] Hoagland DR, Arnon DI (1950) The water-culture method for growing plants without soil. Circular California Agricultural Experiment Station 347.

[28] Schmutz J, McClean PE, Mamidi S, Wu GA, Cannon SB, Grimwood J et al (2014) A reference genome for common bean and genome-wide analysis of dual domestications. Nature Genetics 46: 707–713. 10.1038/ng.3008 24908249 PMC7048698

[29] Langmead B, Trapnell C, Pop M, Salzberg SL (2009) Ultrafast and memory-efficient alignment of short DNA sequences to the human genome. Genome Biology 10: R25 10.1186/gb-2009-10-3-r25 19261174 PMC2690996

[30] Trapnell C, Pachter L, Salzberg SL (2009) TopHat: discovering splice junctions with RNA-Seq. Bioinformatics 25: 1105–1111. 10.1093/bioinformatics/btp120 19289445 PMC2672628

[31] Huang DW, Sherman BT, Lempicki RA (2008) Systematic and integrative analysis of large gene lists using DAVID bioinformatics resources. Nature Protocols 4: 44–57. 10.1038/nprot.2008.211 19131956

[32] Trapnell C, Williams BA, Pertea G, Mortazavi A, Kwan G, van Baren M, et al (2010) Transcript assembly and quantification by RNA-Seq reveals unannotated transcripts and isoform switching during cell differentiation. Nature Biotechnology 28: 511–515. 10.1038/nbt.1621 20436464 PMC3146043

[33] Trapnell C, Hendrickson DG, Sauvageau M, Goff L, Rinn JL, Pachter L (2013) Differential analysis of gene regulation at transcript resolution with RNA-seq. Nature Biotechnology 31: 46–53. 10.1038/nbt.2450 23222703 PMC3869392

[34] Astudillo C, Fernandez AC, Blair MW, Cichy KA (2013) The Phaseolus vulgaris ZIP gene family: identification, characterization, mapping, and gene expression. Frontiers in Plant Science 4. 10.3389/fpls.2013.00286 PMC3726863 23908661

[35] Li H, Handsaker B, Wysoker A, Fennell T, Ruan J, Homer N et al (2009) The sequence alignment/map format and SAMtools. Bioinformatics 25: 2078–2079. 10.1093/bioinformatics/btp352 19505943 PMC2723002

[36] Sankaran RP, Huguet T, Grusak MA (2009) Identification of QTL affecting seed mineral concentrations and content in the model legume Medicago truncatula. Theoretical and Applied genetics 119: 241–253. 10.1007/s00122-009-1033-2 19396421

[37] Cichy KA, Caldas GV, Snapp SS, Blair MW (2009) QTL analysis of seed iron, zinc, and phosphorus levels in an Andean bean population. Crop Science 49: 1742–1750.

[38] Gelin J, Forster S, Grafton K, McClean P, Rojas-Cifuentes G (2007) Analysis of Seed Zinc and Other Minerals in a Recombinant Inbred Population of Navy Bean (L.). Crop Science 47: 1361–1366.

[39] Pinheiro C, Baeta JP, Pereira AM, Domingues H, Ricardo CP (2010) Diversity of seed mineral composition of Phaseolus vulgaris L. germplasm. Journal of Food Composition and Analysis 23: 319–325.

[40] Cakmak I, Pfeiffer WH, McClafferty B (2010) Review: Biofortification of durum wheat with zinc and iron. Cereal Chemistry 87: 10–20.

[41] Oliker M, Poljakoff-Mayber A, Mayer A (1978) Changes in weight, nitrogen accumulation, respiration and photosynthesis during growth and development of seeds and pods of Phaseolus vulgaris. American Journal of Botany: 366–371.

[42] Bhakta MS, Jones VA, Vallejos CE (2015) Punctuated Distribution of Recombination Hotspots and Demarcation of Pericentromeric Regions in Phaseolus vulgaris L. PloS One 10: e0116822 10.1371/journal.pone.0116822 25629314 PMC4309454

[43] Berger S, Bell E, Sadka A, Mullet JE (1995) Arabidopsis thaliana Atvsp is homologous to soybean VspA and VspB, genes encoding vegetative storage protein acid phosphatases, and is regulated similarly by methyl jasmonate, wounding, sugars, light and phosphate. Plant Molecular Biology 27: 933–942. 7766883 10.1007/BF00037021

[44] Arondel V, Vergnolle C, Cantrel C, Kader J-C (2000) Lipid transfer proteins are encoded by a small multigene family in *Arabidopsis thaliana* . Plant Science 157: 1–12. 10940464 10.1016/s0168-9452(00)00232-6

[45] Rashid A, Badhan A, Deyholos M, Kav N (2013) Proteomic profiling of the aleurone layer of mature Arabidopsis thaliana seed. Plant Molecular Biology Reporter 31: 464–469.

[46] Chappell J, Chrispeels MJ (1986) Transcriptional and posttranscriptional control of phaseolin and phytohemagglutinin gene expression in developing cotyledons of *Phaseolus vulgaris* . Plant Physiology 81: 50–54. 16664806 10.1104/pp.81.1.50 PMC1075281

[47] Gepts P, Osborn T, Rashka K, Bliss F (1986) Phaseolin-protein variability in wild forms and landraces of the common bean (Phaseolus vulgaris): evidence for multiple centers of domestication. Economic Botany 40: 451–468.

[48] Debouck DG, Toro O, Paredes OM, Johnson WC, Gepts P (1993) Genetic diversity and ecological distribution ofPhaseolus vulgaris (Fabaceae) in North western South America. Economic Botany 47: 408–423.

[49] Bobb AJ, Eiben HG, Bustos MM (1995) PvAlf, an embryo-specific acidic transcriptional activator enhances gene expression from phaseolin and phytohemagglutinin promoters. The Plant Journal 8: 331–343. 7550372 10.1046/j.1365-313x.1995.08030331.x

[50] Sheokand S, Dahiya P, Vincent J, Brewin N (2005) Modified expression of cysteine protease affects seed germination, vegetative growth and nodule development in transgenic lines of Medicago truncatula. Plant Science 169: 966–975.

[51] Grotz N, Fox T, Connolly E, Park W, Guerinot ML, Eide D (1998) Identification of a family of zinc transporter genes from Arabidopsis that respond to zinc deficiency. Proceedings of the National Academy of Sciences 95: 7220–7224. 10.1073/pnas.95.12.7220 PMC22785 9618566

[52] Li S, Zhou X, Huang Y, Zhu L, Zhang S, Zhao Y, et al (2013) Identification and characterization of the zinc-regulated transporters, iron-regulated transporter-like protein (ZIP) gene family in maize. BMC Plant Biology 13: 114 10.1186/1471-2229-13-114 23924433 PMC3751942

[53] Kim SA, Punshon T, Lanzirotti A, Li L, Alonso JM, Ecker JR, et al (2006) Localization of iron in Arabidopsis seed requires the vacuolar membrane transporter VIT1. Science 314: 1295–1298. 17082420 10.1126/science.1132563

[54] Stephan UW, Scholz G (1993) Nicotianamine: mediator of transport of iron and heavy metals in the phloem? Physiologia Plantarum 88: 522–529.

[55] Hell R, Stephan UW (2003) Iron uptake, trafficking and homeostasis in plants. Planta 216: 541–551. 12569395 10.1007/s00425-002-0920-4

[56] Guo W-J, Meetam M, Goldsbrough PB (2008) Examining the specific contributions of individual Arabidopsis metallothioneins to copper distribution and metal tolerance. Plant Physiology 146: 1697–1706. 10.1104/pp.108.115782 18287486 PMC2287344

[57] Ren Y, Liu Y, Chen H, Li G, Zhang X, Zhao J (2012) Type 4 metallothionein genes are involved in regulating Zn ion accumulation in late embryo and in controlling early seedling growth in Arabidopsis. Plant, Cell & Environment 35: 770–789. 10.1111/j.1365-3040.2011.02450.x 22014117

[58] Blair MW, Medina JI, Astudillo C, Rengifo J, Beebe SE, Machado G, et al (2010) QTL for seed iron and zinc concentration and content in a Mesoamerican common bean (*Phaseolus vulgaris* L.) population. Theoretical and Applied Genetics 121: 1059–1070. 10.1007/s00122-010-1371-0 20532862

[59] Blair MW, Astudillo C, Grusak MA, Graham R, Beebe SE (2009) Inheritance of seed iron and zinc concentrations in common bean (Phaseolus vulgaris L.). Molecular Breeding 23: 197–207.

[60] Hara M, Fujinaga M, Kuboi T (2005) Metal binding by citrus dehydrin with histidine-rich domains. Journal of Experimental Botany 56: 2695–2703. 16131509 10.1093/jxb/eri262

[61] Sankaran RP, Grusak MA (2014) Whole shoot mineral partitioning and accumulation in pea (Pisum sativum). Frontiers in Plant Science 5. 10.3389/fpls.2014.00149 PMC4006064 24795736

