# Supplementary material for: Transcriptome Characterization of Developing Bean (Phaseolus vulgaris L.) Pods from Two Genotypes with Contrasting Seed Zinc Concentrations
Source: PLoS One. 2015 Sep 14;10(9):e0137157. doi: 10.1371/journal.pone.0137157 (PMC4569411; doi:10.1371/journal.pone.0137157)
Supplement: S1 File — Table A. Comparison of fold change of PvZIP and Pv bZIP obtained from RNA-seq and qRT PCR validation of PvZIP and Pv bZIP genes in developing pods. Correlation between normalized sequencing counts in RNA-seq and expression determined by RT qPCR showed high correlation between two methodologies (r = 0.85 p = 0.05). Table B. Primer list for gene expression analysis via RT-qPCR. Four member of the ZIP gene family and two member of the bZIP family were amplified via RT-qPCR to validate the expression profiles obtained by RNA-seq. (DOCX) [file pone.0137157.s001.docx]

Transcriptome Characterization of developing bean (*Phaseolus vulgaris* L.) pods from two genotypes with contrasting seed zinc concentrations.

Carolina Astudillo, Department of Plant, Soil and Microbial Sciences, Michigan State University, 1066 Bogue St., East Lansing, MI 48824

Andrea C. Fernandez, Department of Plant, Soil and Microbial Sciences, Michigan State University, 1066 Bogue St., East Lansing, MI 48824

Karen A Cichy, USDA-ARS, Sugarbeet and Bean Research Unit and Department of Plant, Soil and Microbial Sciences, Michigan State University, 1066 Bogue St., East Lansing, MI 48824

Corresponding Author: Karen Cichy ([Karen.Cichy@ars.usda.gov](mailto:Karen.Cichy@ars.usda.gov))

**Validation of the RNA-seq results with quantitative RT-PCR.**

**The manuscript Astudillo et al., (2013) includes RT-PCR for some of the zinc transporter genes highlighted in the RNA seq study. Detailed methods can be found in that paper. Direct comparison of RT-PCR and RNA seq results are found in Table A and primers used for RT PCR are found in Table B. The citation for this work is:** *Astudillo, C., Fernandez, A., Blair, M., Cichy, K.A. Phaseolus vulgaris ZIP gene family: identification, characterization, mapping and gene expression. Frontiers in Plant Science. 4:286. 2013.*

*http://journal.frontiersin.org/article/10.3389/fpls.2013.00286/abstract*

**Table A**. Comparison of fold change of PvZIP and Pv bZIP obtained from RNA-seq and qRT PCR validation of PvZIP and Pv bZIP genes in developing pods. Correlation between normalized sequencing counts in RNA-seq and expression determined by RT qPCR showed high correlation between two methodologies (r=0.85 p=0.05).

|  | **Gene** | **RT qPCR** | **RNA-seq** |
| --- | --- | --- | --- |
| **Transcript** |  | **G19833 vs Dor 364 (fold change)** | **Albion vs Voyage (fold change)** |
| Phvul.005G034400 | Pv bZIP1 | 1.2 | 0.0 |
| Phvul.011G035700 | Pv bZIP2 | 0.6 | 0.2 |
| Phvul.002G099700 | PvZIP2 | 0.1 | 0.1 |
| Phvul.005G149800 | PvZIP9 | 0.0 | 0.0 |
| Phvul.005G048900 | PvZIP8 | 0.0 | 0.4 |
| Phvul.006G003300 | PvZIP12 | 2.6 | 2.2 |
| Phvul.006G070200 | PvZIP13 | 0.8 | 0.2 |
| Phvul.009G077700 | PvIRT | 0.0 | 0.0 |

**Table B.** Primer list for gene expression analysis via RT-qPCR. Four member of the ZIP gene family and two member of the bZIP family were amplified via RT-qPCR to validate the expression profiles obtained by RNA-seq

| **Gene** | **Forward primer** | **Reverse primer** |
| --- | --- | --- |
| PvZIP8 | GGTGTTGCTGTTGCACTTCC | GCGAAGCCAGAAAGTGTTGC |
| PvZIP12 | GGGCAGAGGCAAGTGCAGGG | GGGCGTGATGGAGATGCAGGA |
| PvZIP13 | CGCGCTCTTCGATTGCCAGGT | CCACCGGCGTGTAGTGCGTA |
| PvIRT3 | AGAATAACACCATCCCCAAAATTA | AGTCACTATGGGAATGTCACAGAA |
| bZIP1 | ATGCAACCCACCTGGCCCTGATGCT | TGCCTGCCCTTGTAGTTTCCTCGCT |
| bZIP2 | ATCGGGAGAAGAAGAAGGCTCGCGC | TCCGGCCCCTTATGTCCACCAGCAA |
| PvactinII | TGCCATCCAGGCCGTTCTTTCA | GGGGACTGTGTGGCTGACACC |

**PvZIP**: Zrt and irt-like protein in *Phaseolus vulgaris*; members 8, 12, 13 and PvIRT member 3.

**bZIP**: transcription factors that regulate ZIP genes.

**PvactinII** was used as reference gene
